# Difficult-to-treat resistant Gram-negative bacteria and genomic resemblances between colonization and infection among patients in an intensive care unit of a tertiary care hospital in Bangladesh

**DOI:** 10.1128/spectrum.00121-26

**Published:** 2026-07-23

**Authors:** Gazi Md. Salahuddin Mamun, Md. Shahinur Rahaman, Sanzida Khan, Md. Aminul Islam, Shabrina Sharmin, Dilruba Ahmed, Debashis Sen, Mohammad Jubair, Tazkia Ahmed, Md. Ali Amin Nabin, Kabid Ahmed, Syeda Mah-E-Muneer, Aninda Rahman, Md. Mozaffer Hossain, Mustafizur Rahman, Fahmida Chowdhury

**Affiliations:** 1 Infectious Diseases Division, International Centre for Diarrhoeal Disease Research, Bangladesh (icddr,b), Dhaka, Bangladesh; 2 Clinical and Diagnostic Services, International Centre for Diarrhoeal Disease Research, Bangladesh (icddr,b), Dhaka, Bangladesh; 3 Genome Centre, International Centre for Diarrhoeal Disease Research, Bangladesh (icddr,b), Dhaka, Bangladesh; 4 School of Clinical Medicine, Faculty of Medicine and Health, University of New South Wales, Sydney, Australia; 5 Directorate General of Health Services, Ministry of Health and Family Welfare, Dhaka, Bangladesh; 6 Department of Anaesthesia, Pain, Palliative & Intensive Care, Dhaka Medical College Hospital, Dhaka, Bangladesh; 7 Department of Anaethesia & Intensive Care, Gonoshasthaya Somaj Vittik Medical College, Dhaka, Bangladesh; Universita degli Studi dell'Insubria, Varese, Italy; Zhejiang Provincial People's Hospital, Hangzhou, Zhejiang, China; King Saud University, Riyadh, Saudi Arabia

**Keywords:** difficult-to-treat resistance, Gram-negative bacteria, intensive care unit, colonization, infection

## Abstract

Colonization with difficult-to-treat-resistant Gram-negative bacteria (DTR-GNB) increases the risk of subsequent infections with limited treatment options. This study aimed to assess the burden of DTR-GNB colonization in ICU patients, explore its association with clinical outcomes, and examine genomic similarities. This secondary analysis included patients enrolled within 24 h of ICU admission between July 2023 and January 2024. Rectal swabs were collected at enrollment, on days 3, 7, and weekly during ICU stay to detect colonization. Bacterial isolates grown on selective chromogenic agar media were identified and tested for antimicrobial susceptibility using matrix-assisted laser desorption ionization-time of flight mass spectrometry (MALDI-TOF MS) and automated broth microdilution, respectively. Blood, urine, and/or tracheal aspirate cultures were performed if clinically suspected sepsis. Whole-genome sequencing (WGS) was performed on paired colonization and infection isolates, and genomic relatedness was assessed using FastANI, core-genome single-nucleotide polymorphism (SNP) analysis, and phylogenetic reconstruction. Among 373 patients, 181 (48.5%) were colonized with DTR-GNB; 76 (20.4%) at enrollment, and 105 (53.0%) acquired during hospital stay. Among 52 (13.9%) patients evaluated for suspected infection, 30 (57.7%) had positive cultures, predominantly *Acinetobacter baumannii* (*n* = 15) and *Klebsiella pneumoniae* (*n* = 11) of DTR-phenotypes. Compared to non-colonized patients, patients colonized with DTR-GNB had higher risks of infections (risk ratio [RR]: 2.18, 95% CI: 1.27–3.76) and longer ICU stays (median 7 vs 2 days, *P <* 0.001). DTR-GNB-infected patients had a higher risk of death (RR: 1.57, 95% CI: 1.34–1.84) compared to patients without DTR-GNB infection. WGS revealed that 13 of 14 paired colonization-infection isolates were conspecific, with three pairs being highly clonal; whereas the remaining pairs showed greater genomic divergence, consistent with the SNP and phylogenetic analyses. While common, more than half acquired DTR-GNB colonization from the ICU. Its association with subsequent infection and prolonged ICU stays underscores the need for enhanced infection prevention and control measures to mitigate nosocomial transmission and improve patient outcomes.

**IMPORTANCE:**

This study underscores the growing threat posed by difficult-to-treat resistant Gram-negative bacteria (DTR-GNB) in intensive care units. Nearly half of critically ill patients were colonized, with a considerable proportion acquiring these multidrug-resistant organisms during their ICU stay. Colonization with these pathogens substantially increased the risk of subsequent infections, even by the same colonizing strain, prolonged ICU stays, and likely worsened clinical outcomes due to the unavailability of susceptible antibiotics. Alarmingly, more than 90% of patients infected with DTR-GNB expired in the hospital. These findings highlight the urgent need for robust infection prevention and control strategies to curb nosocomial transmission and mitigate the impact of DTR-GNB on vulnerable patient populations. Addressing this emerging resistance phenotype is critical to improving patient safety and reducing the burden on healthcare systems.

## INTRODUCTION

Antimicrobial resistance (AMR) has emerged as one of the leading public health threats of the 21st century and the leading cause of infectious disease-related deaths globally (1). In 2021 alone, bacterial AMR was associated with an estimated five million deaths, with the highest burdens in low-resource settings (1). Among resistant pathogens, Gram-negative bacteria (GNB), including Enterobacterales, *Pseudomonas aeruginosa*, and *Acinetobacter baumannii*, are the most clinically important as they have the capability to cause widespread dissemination, outbreaks, and difficult-to-treat infections due to their plasmid-mediated transfer of resistance and frequent nosocomial transmission (2–4).

Historically, multidrug-resistant and extensively drug-resistant phenotypes have been used to describe severe resistance patterns; however, their correlation with patients’ clinical outcomes has been inconsistent (5–8). More recently, the concept of difficult-to-treat resistance (DTR) has emerged as a clinically meaningful category mostly for clinically important Gram-negative bacteria, defined by resistance to nearly all first- and second-line antibacterial agents, including all β-lactams and fluoroquinolones (2). This classification reflects real-world therapeutic challenges and better predicts adverse clinical outcomes, such as increased mortality and limited treatment options (2). Importantly, global surveillance data indicate that the trend of DTR phenotypes is rising among World Health Organization (WHO) priority bacterial pathogens (9).

The mortality related to DTR is found to be higher in several studies. A multicenter retrospective study in the United States reported that although the prevalence of DTR was 1% among all Gram-negative bacterial bloodstream infections (BSI), the crude mortality was 43%, whereas it was 35% for carbapenem-resistant, 22% for extended-spectrum cephalosporin-resistant, and 18% for fluoroquinolone-resistant BSI (2). Similarly, another surveillance study from Korea observed that the crude mortality for Gram-negative bloodstream infection (GNBSI) caused by DTR was 50% and significantly higher than that in other resistance categories (10). In a study conducted in India, the neighboring country of Bangladesh, they observed a GNBSI incidence of 38%, with 3.9 times increased mortality among patients with DTR GNBSI (11).

In Bangladesh, there is a scarcity of data on DTR infections. Limited studies have documented colonization with multidrug-resistant organisms, including DTR Gram-negative bacteria (DTR-GNB), both among hospitalized patients and community participants (12). While colonization itself is asymptomatic, it represents a critical reservoir for subsequent infection and nosocomial transmission (4, 13–15). Understanding the burden of DTR colonization and its relationship to infection and clinical outcomes is essential to strengthening infection prevention strategies and antimicrobial stewardship.

We aimed to determine the prevalence and acquisition of DTR-GNB colonization among critically ill patients admitted to an intensive care unit (ICU) of a tertiary care hospital in Bangladesh. We also evaluated the association between colonization and subsequent infection and examined the clinical outcomes and genomic relatedness of colonization-infection pathogen pairs.

## MATERIALS AND METHODS

### Study design, site, and population

This study was done as a secondary analysis of a prospective cohort study that was conducted in an ICU of a tertiary care government hospital in Dhaka city, Bangladesh, during July 2023 to February 2024. The hospital serves as a referral center for under-resourced populations, with most admitted patients coming from low- or lower-middle-income families across the country. The study ICU has a capacity of 32 beds and admits patients from varying disciplines and diagnoses, such as post-operative complications requiring ICU management, head injury, road traffic accident, cerebrovascular disease, encephalitis, severe respiratory distress with heart failure, expanded dengue syndrome, septic shock, etc. All patients, regardless of sex or age, admitted to the ICU, were eligible for the study if they or their legal guardian provided written informed consent. Patients having severe neutropenia (absolute neutrophil count < 500/mm^3^), gastrointestinal bleeding, or who refused to follow up were excluded.

### Patient enrollment and sample collection

Patients were enrolled in this study by study physicians within 24 h of their admission to the ICU. At enrollment, a rectal swab sample was collected by trained study staff from all study patients. Follow-up rectal swabs were collected on days 3, 7, and 14, and then at 7-day intervals until in-hospital outcome (death, discharge from the ICU, or transfer to a high dependency unit within the same hospital). Sterile COPAN eSwab sticks containing liquid Amies media were used for collecting rectal swabs. Sociodemographic information was collected at enrollment from the patients or their caregivers, if the patient was unable to speak. Clinical and laboratory data were collected both at enrollment and during each follow-up. In addition to study physicians, patients were monitored daily by clinicians, and clinical samples (e.g., blood, tracheal aspirate, or urine) were collected as clinically indicated at any time during their ICU stay. Clinical samples were collected in BACT/ALERT FA Plus containers by trained hospital staff. After collection, samples were immediately placed in a cool box maintained at 2°C–8°C and transported to the Clinical Microbiology Laboratory of icddr,b on the same day for processing.

### Laboratory procedure

All collected specimens were tested at the Clinical Microbiology Laboratory of icddr,b on the same day of collection. The rectal swabs were plated on both ESBL and mSuperCARBA CHROMagar media for any growth. Thus, we didn’t screen any organisms that were susceptible to extended-spectrum cephalosporins and/or carbapenems. This selective chromogenic agar can detect *Escherichia coli* as dark pink or reddish colonies, *Klebsiella*, *Enterobacter*, and *Citrobacter* as metallic blue colonies, *Pseudomonas* as translucent cream or blue colonies, and *Acinetobacter* as opaque cream colonies (https://www.chromagar.com/en/product/chromagar-msupercarba) (16). The clinical samples were incubated in the BacT/ALERT 3D machine and checked every 6 h for any positive growth for up to 5 days. All positive growths from selective chromogenic agar media or BacT/ALERT 3D machine were then tested for identification and antibiotic susceptibility by matrix-assisted laser desorption ionization-time of flight mass spectrometry (MALDI-TOF MS, Vitek MS Prime) and automated broth microdilution (Vitek 2), respectively. Antibiotic susceptibility testing (AST) was performed according to the 2023 Clinical and Laboratory Standards Institute guidelines using Vitek 2 AST-N405 cards that can test against specific antibiotics designed for Gram-negative bacteria.

### Whole-genome sequencing

Among the 30 culture-confirmed infections, 13 patients had 14 paired isolates of bacterial species recovered from both clinical (blood, urine, and tracheal aspirate) and rectal swab samples of the same patients. All of these available paired isolates underwent WGS to detect the genomic similarities between colonized and infected strains found from the same patients. Genomic DNA was extracted from these bacterial isolates using the Qiagen DNeasy Blood and Tissue Kit (Qiagen, catalog no. 69504) with modifications from the CDC PulseNet Total DNA Extraction Protocol. Bacterial pellets were centrifuged at 14,000 rpm for 5 min, and DNA quality and concentration were assessed using a NanoDrop spectrophotometer (260/280 ~1.8, 260/230 ~2.0) and a Qubit 4.0 Fluorometer, confirming suitability for library preparation. Libraries were prepared using the Illumina DNA Prep Kit (catalog no. 20060059) on an automated liquid handler (epMotion 5075; Eppendorf). Pooled libraries were quantified, diluted, denatured, and loaded onto the NextSeq 2000 platform using the NextSeq 1000/2000 P1 Reagent Cartridge XLEAP-SBS (Illumina) for paired-end 2 × 150 bp sequencing, generating high-resolution whole-genome data for downstream comparative analysis.

### Genomic analysis

Raw paired-end reads were processed using the Phoenix pipeline v2.1.1 (https://github.com/CDCgov/phoenix). Adapter trimming and low-quality base removal were performed using the tool Fastp v0.23.4 (https://github.com/OpenGene/fastp) (Phred ≥ 15), followed by quality assessment using the tool FastQC v0.12.1 (https://github.com/s-andrews/FastQC), evaluating per-base quality, GC content, and adapter contamination. *De novo* genome assembly was performed using the tool SPAdes v4.2.0 (https://github.com/ablab/spades) with BayesHammer error correction and iterative k-mer sizes (21, 33, 55), retaining contigs ≥500 bp to optimize assembly integrity. Species-level identification was confirmed using the tool Kraken2 v2.1.6 (https://github.com/DerrickWood/kraken2) with the standard database, and strain-level typing was conducted using the tool MLST v2.23.0 (https://github.com/tseemann/mlst) supplemented with a custom MLST database for enhanced specificity.

Comparative genomic analyses of infection and colonization isolates were performed using FastANI v1.34 (https://github.com/ParBLiSS/FastANI) to calculate pairwise average nucleotide identity (ANI), with values exceeding 95% indicating high genomic relatedness. Variant calling was conducted using Snippy v4.6.0 (https://github.com/tseemann/snippy), which aligned reads to species-specific reference genomes with BWA-MEM and identified variants using FreeBayes (minimum depth ≥10×, Phred quality >20). The tool snippy-core was then used to generate a concatenated alignment of all core single-nucleotide polymorphisms (SNPs) shared across samples, providing the total number of conserved variant positions and core SNP counts for the quantification of strain-level genetic differences between infection and colonization isolates to assess within-host evolutionary variation. Differences in core SNP profiles between infection and colonization groups were examined to highlight genetic variants. Additionally, species-specific phylogenetic trees were reconstructed for paired *Klebsiella pneumoniae* and *A. baumannii* isolates. Core-genome alignments were generated using snippy-core, and maximum-likelihood phylogenies were inferred using IQ-TREE with ModelFinder and 1,000 SH-aLRT and 1,000 ultrafast bootstrap replicates. Trees were visualized and annotated in iTOL. Phylogenetic reconstruction was not performed for *E. coli* and *P. aeruginosa*, as only one paired isolate set was available for each species.

### Operational definitions

We analyzed the four major and clinically important WHO priority Gram-negative bacteria in our study: *E. coli*, *K. pneumoniae*, *A. baumannii*, and *P. aeruginosa* (9, 10). To characterize combined resistance patterns, we applied the DTR definition to all isolates of these four Gram-negative bacteria. DTR was defined as non-susceptibility (intermediate or resistant) to all tested beta-lactams (including penicillin, cephalosporins, carbapenems, and monobactams) and fluoroquinolone antibiotics (2, 9, 10). In our study, we tested against amoxicillin-clavulanic acid, cefuroxime, ceftriaxone, cefepime, cefoperazone-sulbactam, meropenem, imipenem, ertapenem, piperacillin-tazobactam, and ciprofloxacin for all our studied Gram-negative bacteria. Colonization was defined as the presence of positive growth from the rectal swab culture (17, 18). Those who had clinical signs and symptoms of infection and clinical samples (blood, urine, and/or tracheal aspirate) sent for culture were considered suspected infection. Among those, patients with positive growth in culture from any clinical sample(s) were categorized as confirmed infection. Any underlying disease condition present among the patients during their hospital stay was considered a comorbidity, such as surgical wound infection, diabetes mellitus, chronic pulmonary disease, malignancy, cardiovascular disease, etc.

### Sample size

This secondary analysis was based on a previous study that enrolled 373 individuals. All enrolled patients of that study were included in this analysis.

### Data management and statistical analysis

Study physicians collected the data into a pre-tested semi-structured paper questionnaire and then entered it daily into an electronic cloud system via tablet. Descriptive statistics were used to summarize sociodemographic, clinical, laboratory, and other relevant data. Medians with interquartile ranges (IQRs) were calculated for skewed continuous variables and differences using the Wilcoxon Rank Sum test. Log-binomial regression analyses were performed for the risk analysis of categorical variables. Considering a 95% confidence interval, we considered a *P* value < 0.05 as statistically significant. Data management and statistical analyses were performed using STATA SE (StataCorp LP, College Station, TX, 2017, version 15). Upset plots were done using Python (version 3.12). Lollipop and dot plots were done using R Studio (version 2025.09.1).

## RESULTS

### Enrollment and follow-ups

**Fig 1:**
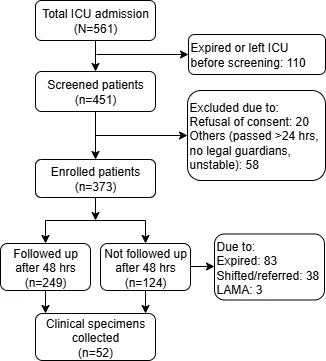
Flowchart detailing patients’ enrollment and longitudinal follow-up during July 2023 to February 2024.

A total of 373 patients were enrolled in this study, of which 249 (66.8%) completed at least one follow-up after 48 h of hospital stay (Fig. 1). The remaining patients expired, were shifted to other wards, left against medical advice (LAMA), or referred outside from the ICU within 48 h of enrollment.

### Baseline characteristics

The median age of the patients was 40 years (IQR: 24–50), and 235 (63%) were male (Table 1). Sixty-three percent of the patients resided in rural areas, and 78.8% had visited a healthcare facility at least once in the year preceding hospitalization. Ninety-five percent of patients had at least one comorbidity, with surgical wound being the most common (60.6%). All patients received at least one antibiotic within 1 month prior to admission. The average median duration of hospital stay was 9 days before being transferred to the ICU (Table 1).

**TABLE 1:** Baseline descriptive characteristics of the study patients enrolled from the ICU of a tertiary care hospital in Bangladesh, from July 2023 to February 2024

Characteristics	Patients, *N* = 373 *n* (%)
Age in years (median, IQR)	40 (24–50)
Male	235 (63.0)
Rural residence	234 (62.7)
Any comorbidity	355 (95.2)
Surgical wound	215 (60.6)
Pneumonia	58 (16.3)
DM	51 (14.3)
Visited healthcare facility within 1 year	294 (78.8)
Received antibiotics within 1 month	
No	0
One/two antibiotic(s)	81 (21.7)
>Two antibiotics	292 (78.3)
Admitted from another hospital or ward	293 (78.6)
Hospital length of stay (days) before ICU admission (median, IQR)	9 (5–15)

### Colonization with Gram-negative bacteria

Of the 373 enrolled patients, 304 (81.5%) were colonized with any of the four major Gram-negative bacteria: 247 (66.2%) with *E. coli*, 162 (43.4%) with *K. pneumoniae*, 102 (27.3%) with *A. baumannii*, and 24 (6.4%) with *P. aeruginosa*. Colonization exclusively with *E. coli* was observed among 88 (23.6%) patients. Co-colonization with *E. coli* and *K. pneumoniae* occurred in 80 (21.4%) patients, while 34 (9.1%) patients were colonized with all three bacteria except *P. aeruginosa.* Seven (1.9%) patients were colonized with all four bacteria. Additional colonization combinations, irrespective of their resistance pattern, are illustrated in the upset plot (Fig. S1).

### Difficult-to-treat resistant phenotypes

**Fig 2:**
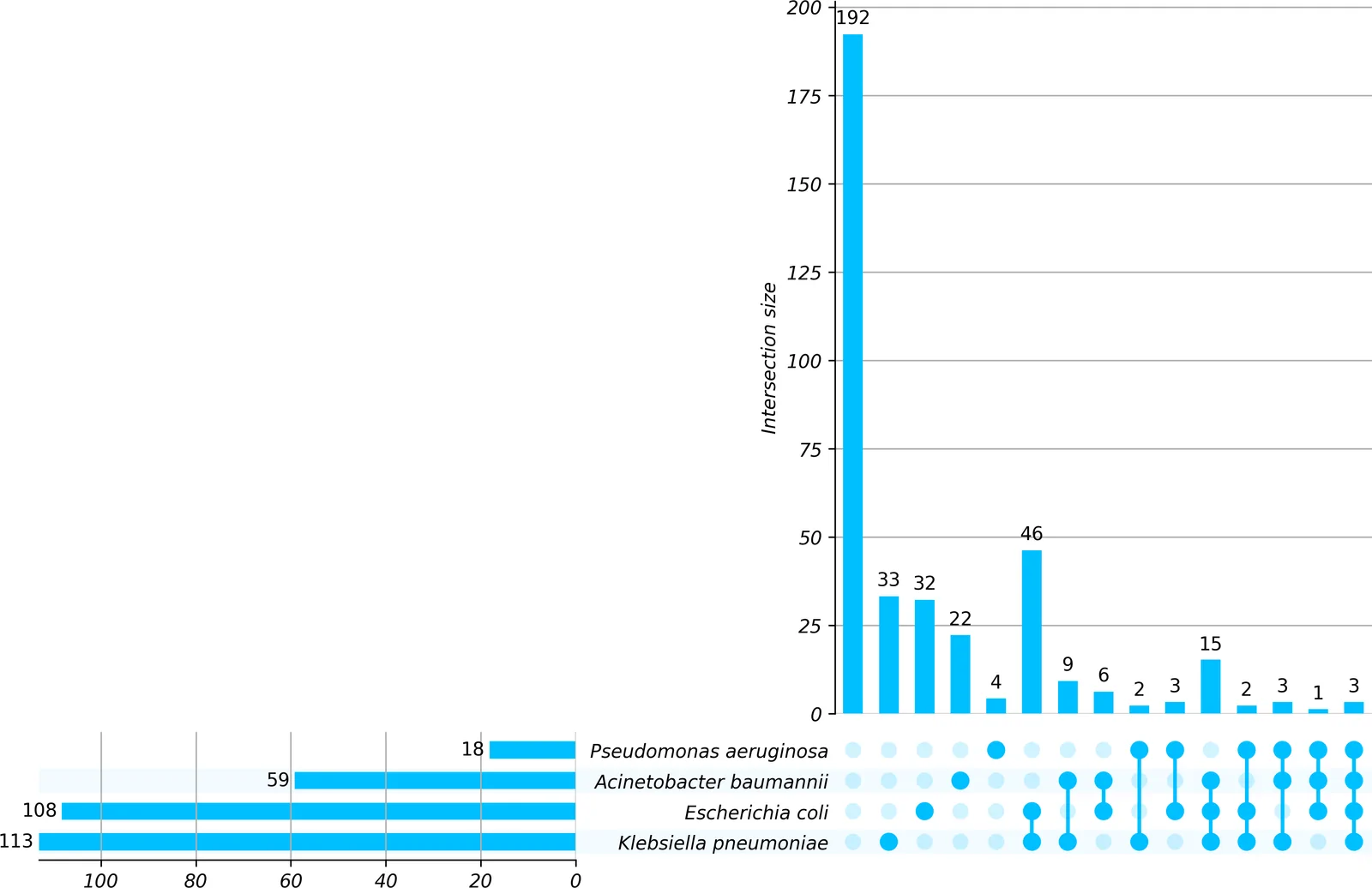
Distribution of enrolled patients colonized with DTR-phenotypes of Gram-negative bacteria (*n* = 373). The horizontal bars show the number of difficult-to-treat-resistant Gram-negative bacteria detected from rectal swabs of the enrolled patients. The vertical bars show the co-colonization with these DTR-GNBs.

A total of 181 (48.5%) patients were colonized with at least one DTR phenotype of GNB. The most prevalent DTR phenotype of GNB was *K. pneumoniae* (113, 30.3%), followed by *E. coli* (108, 29.0%). Difficult-to-treat resistant *A. baumannii* and *P. aeruginosa* were detected among 59 (15.8%) and 18 (4.8%) patients, respectively. Co-colonization with DTR *K. pneumoniae* and *E. coli* occurred in 46 (12.3%) patients, while three patients (0.8%) were colonized with DTR phenotypes of all four GNB (Fig. 2).

### Onset of DTR colonization

**Fig 3:**
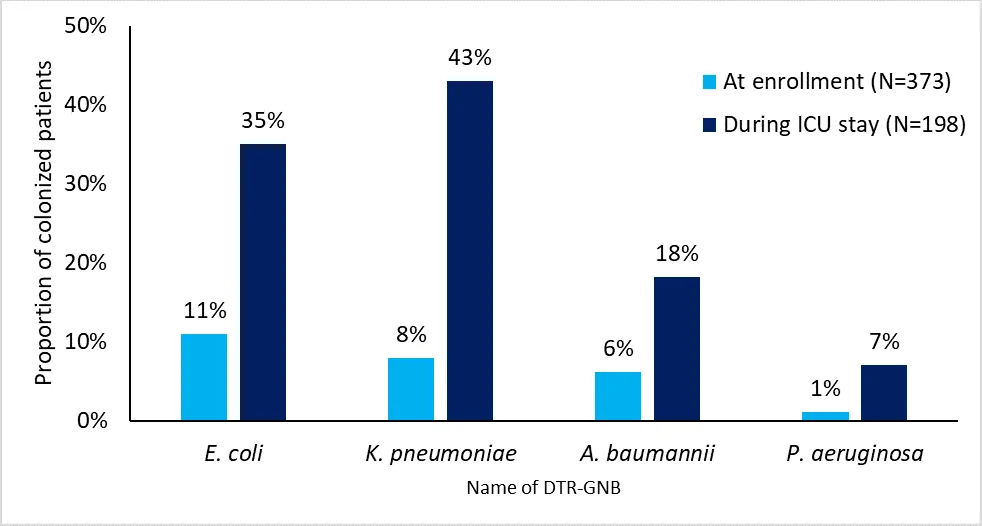
Distribution of the onset of colonization at enrollment and during ICU stay among the patients with DTR-GNB.

At enrollment, 76 (20.4%) patients were colonized with at least one DTR-GNB. DTR *E. coli* was found among 39 (10.5%) patients, *K. pneumoniae* among 28 (7.5%) patients, *A. baumannii* among 23 (6.2%) patients, and *P. aeruginosa* among 4 (1.1%) patients (Fig. 3).

Among the remaining 198 patients who were not colonized with DTR-GNB at enrollment and had at least one follow-up, 105 (53.0%) acquired DTR-GNB colonization during their hospital stay. During their ICU stay, 85 (42.9%) patients acquired *K. pneumoniae*, 69 (34.8%) acquired *E. coli*, 36 (18.2%) acquired *A. baumannii*, and 14 (7.1%) acquired *P. aeruginosa* (Fig. 3).

### Bacterial isolates, colonization, and resistance profiles

A total of 502 *E. coli* isolates were recovered from both selective chromagar culture media, of which 174 (34.7%) exhibited a DTR phenotype. Similarly, 58.6% of *K. pneumoniae* isolates (164 out of 280), 50.0% of *A. baumannii* isolates (76 out of 152), and 73.1% of *P. aeruginosa* isolates (19 out of 26) were DTR phenotypes. Resistance patterns against tested antibiotics of these Gram-negative bacterial isolates are presented in Fig. S2 to S5.

### Clinical infections

Fifty-two (13.9%) patients had suspected clinical infections. From these patients, 49 blood, 21 urine, and 17 tracheal aspirate specimens were collected for culture and susceptibility tests. Among the tested patients, 30 (57.7%) had positive growth in cultures with pathogenic isolates, and multiple pathogens were isolated in 12 (23.1%) cases. A total of 22 (42.3%) patients were infected with the DTR phenotype of GNB. The most frequently identified bacteria from these clinical samples were *A. baumannii* (16, 30.8%), followed by *K. pneumoniae* (13, 25.0%). Of these, 15 *A. baumannii* and 11 *K. pneumoniae* isolates exhibited a DTR phenotype. All four Gram-negative bacteria isolated from different clinical samples are presented in Table 2 according to their DTR phenotype.

**TABLE 2:** Predominant Gram-negative bacteria detected from clinical samples of the enrolled patients and their difficult-to-treat resistance phenotype, from a tertiary care hospital in Bangladesh, from July 2023 to February 2024

Bacterial isolates	Blood *n* = 49 DTR/total	Urine *n* = 21 DTR/total	Tracheal aspirate *n* = 17 DTR/total	Total *n* = 52	Difficult-to-treat resistance *n* (%)
*Acinetobacter baumannii*	9/9	1/1	5/6	16	15 (93.8)
*Klebsiella pneumoniae*	3/4	0	8/9	13	11 (84.6)
*Pseudomonas aeruginosa*	0	1/1	3/3	4	4 (100)
*Escherichia coli*	0	1/1	0/1	2	1 (50.0)

### Association with outcomes

Among the DTR-GNB colonized patients, 35 (19.3%) had infections (19 were culture-confirmed). Among the non-colonized, 17 (8.9%) had infections (11 were culture-confirmed). Log-binomial regression analysis found that patients colonized with DTR-GNB had higher risks of infections (risk ratio [RR]: 2.18, 95% CI: 1.27–3.76). The median duration of ICU stays was longer in colonized patients compared to non-colonized patients (7 days [IQR: 3–14 days] vs 2 days [IQR: 1–5 days], *P <* 0.001). Most of the study patients (223, 59.8%) died in hospital. Among them, 107 (48.0%) were colonized with at least one DTR-GNB. However, no significant difference in death was observed between DTR-GNB colonized and non-colonized groups (Table 3).

**TABLE 3:** Outcome of patients colonized with difficult-to-treat resistance phenotype of Gram-negative bacteria at any time point during ICU stay

Outcomes	DTR-GNB colonized (*n* = 181) *n* (%)	DTR-GNB non-colonized (*n* = 192) *n* (%)	RR (95% CI)	*P* value
Infection	35 (19.3)	17 (8.9)	2.18 (1.27–3.76)	0.005
ICU length of stay in days (median, IQR)	7 (3–14)	2 (1–5)		<0.001
In-hospital death	107 (59.1)	116 (60.4)	0.98 (0.83–1.16)	0.798

Among the infected patients, only one patient with DTR *A. baumannii* (received piperacillin and tazobactam combination and linezolid as their last ongoing antibiotics at discharge) and one with DTR *K. pneumoniae* (received meropenem and teicoplanin as their last ongoing antibiotics at discharge) infection survived during their hospital stay. Both had bloodstream infections. The remaining 20 (90.9%) patients with DTR-GNB infection expired in the hospital. Patients with DTR-GNB infection had a significantly higher risk of mortality (RR 1.57, 95% CI: 1.34–1.84) compared to patients without DTR-GNB infection. Patients infected with DTR-GNB had longer ICU stays (median 7 days vs 4 days); however, this difference was not statistically significant.

### Whole-genome sequencing

**Fig 4:**
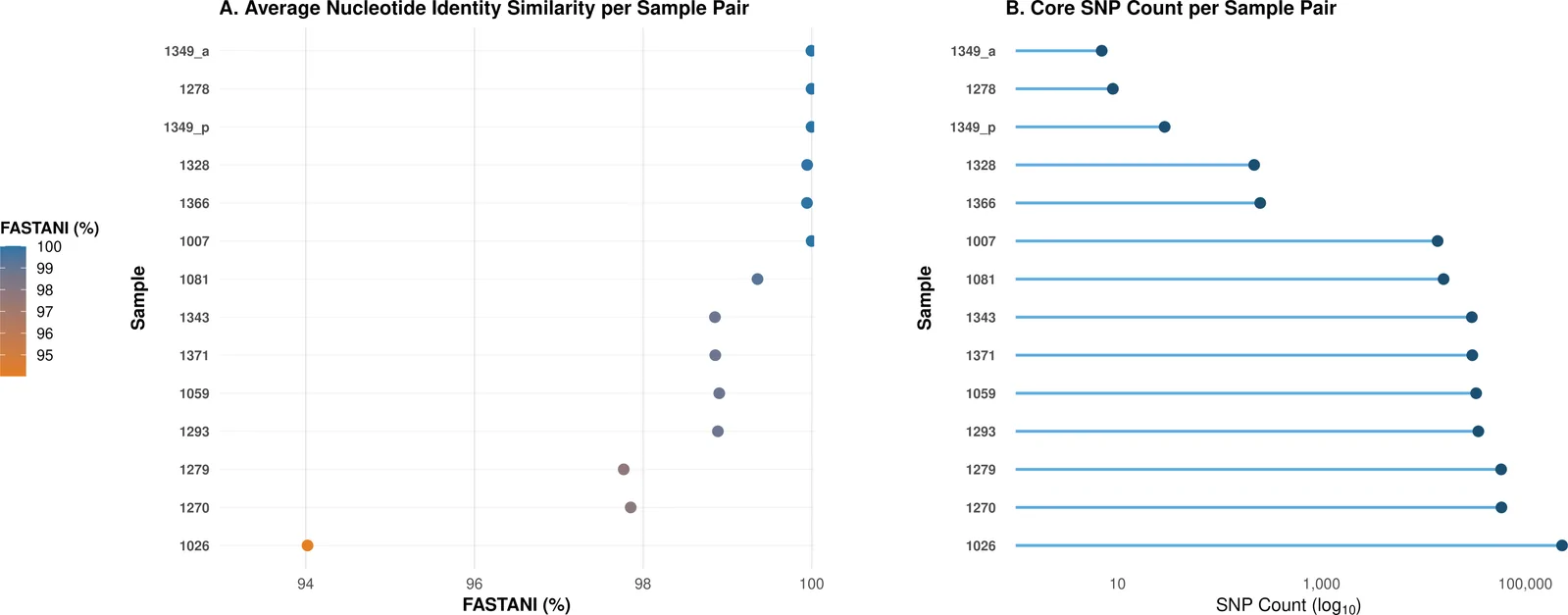
Genomic relatedness between colonization and infection paired isolates. (**A**) Pairwise ANI values for the 14 infection-colonization isolate pairs. Thirteen pairs shared >95% ANI, confirming the same species, while one pair (94.02% ANI) represents a closely related but genetically distinct species. (**B**) Core SNP differences between paired infection and colonization isolates. Isolates with >99% ANI and <50 SNP differences indicate close strain-level relatedness, whereas higher SNP counts (>10,000) reflect greater intra-species variation.

WGS confirmed that 13 of 14 isolate pairs from 12 patients belonged to the same species of *E. coli* (one pair), *K. pneumoniae* (seven pairs), *A. baumannii* (four pairs), and *P. aeruginosa* (one pair), and the remaining pair from another patient was *Klebsiella quasipneumoniae* and *K. pneumoniae* (Table S1). WGS further showed that the paired isolates belonged to diverse sequence types (STs). Among *K. pneumoniae*, several isolates belonged to ST11, whereas the *A. baumannii* isolates comprised multiple STs and novel profiles, highlighting genomic heterogeneity across paired colonization and infection isolates. To assess their genomic relatedness, ANI was calculated using FastANI. Pairwise average nucleotide identity analysis (Fig. 4A) showed that 13 of the 14 isolate pairs shared greater than 95% ANI, confirming species-level identity. Among these, six isolates exhibited more than 99% ANI, indicating close strain-level relatedness. One isolate pair displayed an ANI of 94.02%, slightly below the threshold, suggesting it represents a closely related but distinct species, detected as *K. quasipneumoniae* by WGS, where Vitek MS detected it as *K. pneumoniae*. Pairwise core SNP analysis further characterized genomic variation between colonization and infection isolates (Fig. 4B). Isolates with greater than 99% ANI exhibited correspondingly low core SNP counts, indicating limited genetic divergence and supporting the claim as closely related strains within the same species. Among them, three isolate pairs showed <50 SNP differences, indicating the same strain and transmission from colonization to infection (Table S2). Another two isolate pairs showed up to 251 SNP differences, while the remaining pairs displayed more than 10,000 SNPs, reflecting substantial intra-species variation. Notably, one isolate pair exhibited the highest core SNP count (229,309 SNPs), consistent with its lower ANI (below 95%), supporting its distinct species status. Detailed ANI, genome coverage, assembly length, core SNP data, and sequence types for all isolate pairs are provided in Tables S1 and S2.

Phylogenetic analysis was broadly concordant with the FastANI and SNP comparisons (Fig. S6). Four species were represented among the paired isolate sets (*E. coli*, *A. baumannii*, *K. pneumoniae*, and *P. aeruginosa*), with one additional *Klebsiella quasipneumoniae* colonization isolate detected in one patient. Because only one paired isolate set was available for *E. coli* and *P. aeruginosa*, phylogenetic trees were generated only for *K. pneumoniae* and *A. baumannii*. In both species, infection and colonization isolates were interspersed across the trees rather than forming distinct status-specific clades, and pairs with higher ANI values and lower SNP distances clustered more closely than more divergent pairs, indicating heterogeneous genomic relatedness between paired colonization and infection isolates. The *K. pneumoniae* colonization isolate from one patient was excluded from the phylogeny because it was identified as *K. quasipneumoniae*.

## DISCUSSION

This prospective cohort study found an alarmingly high burden of colonization and infection with DTR-GNB among the critically ill patients in the ICU, which was associated with poor in-hospital outcomes. Evidence of acquisition of clinically important DTR-GNB pathogens during hospital stay underscores the importance of strengthening infection prevention and control measures in high-care settings, which complements antimicrobial stewardship efforts by reducing the incidence of resistant infections.

Almost half of the enrolled patients were colonized with at least one DTR-GNB. Notably, among those initially non-colonized, over half acquired DTR-GNB colonization during their hospital stay, indicating substantial in-hospital transmission and highlighting the burden and spread of multidrug-resistant organisms in this setting.

In this study, *E. coli* was the most frequently identified colonizer at enrollment and *K. pneumoniae* during hospital stay among the difficult-to-treat resistant bacteria, consistent with their known roles as major nosocomial pathogens (19). Nearly one-third of patients were colonized with each of these two predominant DTR organisms. However, no other similar study was found detecting the colonization burden of DTR GNB among the critically ill ICU patients.

Although the most prevalent colonizing isolate (with or without DTR) was *E. coli* (*n* = 502), the highest proportion (73.1%) of DTR phenotype was exhibited in the case of *P. aeruginosa* in our study. A 2019 study in Bangladesh reported that among hospitalized participants, the DTR phenotype was observed in 30% of *E. coli* isolates and 13% of *Klebsiella* spp. isolates (12). Though the prevalence of *E. coli* was similar to our study, we observed a higher prevalence of *K. pneumoniae* in our study among critically ill patients in the ICU. That study enrolled participants from several wards, including medicine, surgery, and gynecology, with only 1% of patients being critically ill and from the ICU. This difference in patient population can explain the higher prevalence observed in our study. However, they didn’t report *P. aeruginosa* or *A. baumannii*.

Among patients with suspected infections who provided clinical specimens, *A. baumannii* and *K. pneumoniae* were detected as the two most predominant pathogens. Alarmingly, almost all of them (91.7% *A. baumannii* and 85.7% *K. pneumoniae*) were non-susceptible to all tested beta-lactams, including carbapenems and fluoroquinolones (DTR phenotype), indicating limited treatment options. As a result, nearly all of them expired in the hospital. Similarly, all *P. aeruginosa* isolates and two-thirds of *E. coli* isolates were found as DTR phenotype among the infected patients. A recent study by Wise et al. stated that DTR phenotypes of Enterobacterales, *P. aeruginosa,* and *A. baumannii* were increasing among clinical isolates globally (9). However, they reported a lower prevalence of DTR phenotypes in the Asia Pacific region than we did in our study: *A. baumannii-calcoaceticus* complex (50%–60% during 2018–2022), Enterobacterales (9.8% in 2020), and *P. aeruginosa* (6%–10% in 2018–2022) (9). The higher prevalence observed in our study can be explained by the fact that, unlike Wise et al., who included Gram-negative bacterial isolates from all hospitalized patients of varying severities, we focused specifically on critically ill ICU patients.

We found that patients colonized with DTR-GNB had a significantly higher risk of infections compared to patients without any infection. Log binomial regression analysis confirmed that colonized patients had 2.18 times higher risk of infection during their hospital stay. Although no study has specifically examined DTR pathogens, our findings are consistent with several other studies reporting that colonization with multidrug-resistant organisms can lead to bloodstream infections (4, 13).

In this study, patients colonized with DTR-GNB experienced prolonged ICU stays (median 7 days vs 2 days) compared to the non-colonized patients, as shown by the Wilcoxon Rank Sum test. However, the higher prevalence of colonization after 48 h in the ICU suggests that the prolonged ICU stay may contribute to its acquisition. Although no study specifically evaluated ICU length of stay among DTR colonized patients, those with DTR infection had longer hospital stays (14 days vs 6 days) compared to patients with nonresistant GNBSI (2). Similarly, Huh K et al. reported that the duration of hospital stay progressively increased with higher degrees of antimicrobial resistance, and patients infected with DTR GNBSI remained hospitalized 2.3 times longer compared to patients without DTR infections (10).

We did not find a significant association between DTR-GNB colonization and mortality in our study. However, the overall death rate was high (59.8%) among the study patients, and many of the enrolled patients (22.3%) died within 48 h of study enrollment, before providing any follow-up samples to detect the presence of healthcare-associated colonization. To the best of our knowledge, no prior study has evaluated the in-hospital outcomes of patients colonized with DTR organisms.

We found significantly higher mortality among DTR-GNB-infected patients than among those who were not infected with DTR-GNB. In our study, the risk of in-hospital death was 1.57 times higher among the DTR-GNB-infected patients. These results are consistent with previous studies that also found increased mortality among DTR-infected patients. A study in the US reported 1.2 times higher risk of death, and another study in Korea reported 3.58 times higher odds of death among patients infected with DTR phenotypes of Gram-negative bacteria (2, 10). Following colonized patients for a longer period, even post-discharge, may reveal an association with mortality, as it allows time for infection with DTR pathogens to occur.

Genomic comparison of paired infection and colonization isolates revealed a high degree of species concordance, with 13 of 14 pairs sharing greater than 95% ANI and 6 isolate pairs exceeding 99% ANI. A higher ANI value indicates greater genomic similarity and typically reflects isolates belonging to the same species (20). Consistently low core SNP counts (up to 251 SNPs) among these pairs further support a strong strain-level relatedness, indicating that infections in most cases likely originated from the patients’ own colonizing strains. A lower number of core SNPs is indicative of higher genetic relatedness between isolate pairs (21). In contrast, one isolate pair (94.02% ANI) exhibited an unusually high core SNP count (229,309 SNPs), suggesting infection by a genetically distinct but closely related lineage, possibly reflecting acquisition from an external source or in-host evolution. The phylogenetic trees were broadly consistent with the FastANI and SNP analyses and provided a complementary visual framework for interpreting genomic relatedness among paired isolates. Importantly, the phylogenetic reconstruction did not show clear separation by clinical status, as infection and colonization isolates were interspersed across the trees rather than forming distinct clades. Taken together, the concordance between FastANI, SNP-based comparisons, and phylogenetic analysis supports the interpretation that the genomic relationship between colonization and infection isolates is heterogeneous, with some pairs being closely related and others representing genetically distinct lineages. The ANI, core SNP analyses, and phylogenetic tree provide genomic evidence linking colonization to subsequent infection and highlight both endogenous and exogenous transmission routes of DTR-GNB in ICU settings. Results from a study among hospitalized children found that intestinal colonization of carbapenem-resistant *K. pneumoniae* was strongly linked to extraintestinal infection (22). These findings reinforce the urgent need for robust infection prevention and control measures to limit the spread of multidrug-resistant pathogens and mitigate their clinical and public health impact in hospitalized and critically ill populations.

A strength of our study is the prospective design, which allowed us to assess both colonization burden and infection in the same cohort and to explore genomic similarities by whole-genome sequencing from admission to in-hospital outcomes. Our study is also strengthened by the use of advanced microbiological testing methods, such as Vitek-2 and Vitek MS Prime, from an ISO 15189:2022 accredited microbiology laboratory and WGS using the NextSeq 2000 platform.

Our study has some limitations. First, as it was conducted in a single intensive care unit, the findings may not be generalizable to critically ill patients in other healthcare settings. Second, we did not collect detailed vital signs or laboratory data from the enrolled patients; therefore, we were unable to apply the illness severity scores like APACHE II or SOFA, or to perform multivariable analyses stratified by disease severity to assess mortality differences. Third, due to budget constraints, environmental sampling was not performed, limiting our ability to determine whether hospital-acquired infections were primarily driven by patient-to-patient transmission or environmental reservoirs. Fourth, although sequence types were identified for the sequenced isolates, resistance gene analysis was not included in the original study design. Therefore, we could not correlate phenotypic resistance profiles with specific genetic determinants or assess the concordance of resistance genes between paired colonization and infection isolates. Fifth, as we used specific chromogenic agar media for the growth of colonized isolates, we might have missed pathogens that were not ESBL- or carbapenemase-producing bacteria. Finally, our lack of post-discharge follow-up limited our ability to assess the long-term impact of DTR colonization, which could be better understood with 6 months to 1 year of prospective follow-up.

### Conclusions

Our study finds a high burden of colonization and infection with the Gram-negative bacteria of difficult-to-treat resistance phenotype among the critically ill patients in the ICU, and an association between colonization and infection. An alarmingly high burden of death among DTR-GNB-infected patients indicates the immediate need for selective antibiotics to treat them effectively to reduce mortality. Notably, most of the colonizations were hospital-acquired, underscoring the need for robust infection prevention and control practices. Also, currently, there is no conclusive and reliable evidence of decolonization practices, especially against Gram-negative bacteria in lower- and middle-income countries having limited resources. Further research should also be planned for evaluating the effectiveness of decolonization strategies, such as selective digestive decontamination or fecal microbiota transplantation, among such high-risk populations with high death rates to reduce the morbidity and mortality related to multidrug-resistant pathogens.

## Supplementary Material

Reviewer comments

## Data Availability

In accordance with the recommendation of its Ethical Review Board, icddr,b does not publicly share personal information of hospitalized patients to ensure privacy and confidentiality. However, data generated from icddr,b’s research projects are available to qualified researchers for secondary data analyses, subject to the approval of a data licensing application and agreement by the icddr,b data center committee of IRB. The data request may be sent to Ms. Shiblee Sayeed, Senior Manager, Research Administration, at shiblee_s@icddrb.org. However, the genomic analysis data are publicly available under the BioProject accession number: PRJNA1457996.

## SUPPLEMENTAL MATERIAL

Supplemental material spectrum.00121-26-s0001.docx Fig. S1 to S6; Tables S1 and S2.

OPEN PEER REVIEW reviewer-comments.pdf An accounting of the reviewer comments and feedback.

ASM does not own the copyrights to Supplemental Material that may be linked to, or accessed through, an article. The authors have granted ASM a non-exclusive, world-wide license to publish the Supplemental Material files. Please contact the corresponding author directly for reuse.
