# Supplementary material for: Difficult-to-treat resistant Gram-negative bacteria and genomic resemblances between colonization and infection among patients in an intensive care unit of a tertiary care hospital in Bangladesh
Source: Microbiol Spectr. 2026 Jul 23;14(9):e00121-26. doi: 10.1128/spectrum.00121-26 (PMC13532269; doi:10.1128/spectrum.00121-26)
Supplement: Supplementary file 1 [file reviewer-comments.pdf]

AMERICAN SOCIETY FOR MICROBIOLOGY | Microbiology Spectrum

Difficult-to-treat Resistant Gram-negative Bacteria and Genomic Resemblances between Colonization and Infection among Patients in an Intensive Care Unit of a Tertiary Care Hospital in Bangladesh

Gazi Md. Salahuddin Mamun, Md. Rahaman, Sanzida Khan, Md. Aminul Islam, Shabrina Sharmin, Dilruba Ahmed, Debashis Sen, Mohammad Jubair, Tazkia Ahmed, Md. Nabin, Kabid Ahmed, Syeda Mah-E-Muneer, Aninda Rahman, Md. Hossain, Mustafizur Rahman, and Fahmida Chowdhury

Corresponding Author(s): Gazi Md. Salahuddin Mamun, International Centre for Diarrhoeal Disease Research Bangladesh

Review Timeline:

Submission Date:	January 12, 2026
Editorial Decision:	April 17, 2026
Revision Received:	May 5, 2026
Accepted:	May 10, 2026

Editor: Gabriele Arcari

Reviewer(s): Disclosure of reviewer identity is with reference to reviewer comments included in decision letter(s). The following individuals involved in review of your submission have agreed to reveal their identity: Yunsong Yu (Reviewer #1); Fawzia E Al-Otaibi (Reviewer #2)

Transaction Report:

(Note: With the exception of the correction of typographical or spelling errors that could be a source of ambiguity, letters and reports are not edited. The original formatting of letters and referee reports may not be reflected in this compilation.)

DOI: https://doi.org/10.1128/spectrum.00121-26

Re: Spectrum00121-26 (Difficult-to-treat Resistant Gram-negative Bacteria and Genomic Resemblances between Colonization and Infection among Patients in an Intensive Care Unit of a Tertiary Care Hospital in Bangladesh)

Dear Dr. Gazi Md. Salahuddin Mamun:

Thank you for the privilege of reviewing your work. Below you will find my comments, instructions from the Spectrum editorial office, and the reviewer comments.

Please return the manuscript within 60 days; if you cannot complete the modification within this time period, please contact me. If you do not wish to modify the manuscript and prefer to submit it to another journal, notify me immediately so that the manuscript may be formally withdrawn from consideration by Spectrum.

Revision Guidelines
To submit your modified manuscript, log into the submission site at https://spectrum.msubmit.net/cgi-bin/main.plex. Go to Author Tasks and click the appropriate manuscript title to begin. The information you entered when you first submitted the paper will be displayed; update this as necessary. Note the following requirements:

• Upload point-by-point responses to the issues raised by the reviewers in a file named "Response to Reviewers," NOT in your cover letter.
• Upload a compare copy of the manuscript (without figures) as a "Marked-Up Manuscript" file.
• Upload a clean .DOC/.DOCX version of the revised manuscript and remove the previous version.
• Each figure must be uploaded as a separate, editable, high-resolution file (TIFF or EPS preferred), and any multipanel figures must be assembled into one file.
• Any supplemental material intended for posting by ASM should be uploaded with their legends separate from the main manuscript. You can combine all supplemental material into one file (preferred) or split it into a maximum of 10 files with all associated legends included.

For complete guidelines on revision requirements, see our Submission and Review Process webpage. Submission of a paper that does not conform to guidelines may delay acceptance of your manuscript.

Data availability: ASM policy requires that data be available to the public upon online posting of the article, so please verify all links to sequence records, if present, and make sure that each number retrieves the full record of the data. If a new accession number is not linked or a link is broken, provide Spectrum production staff with the correct URL for the record. If the accession numbers for new data are not publicly accessible before the expected online posting of the article, publication may be delayed; please contact production staff (Spectrum@asmusa.org) immediately with the expected release date.

Publication Fees: For information on publication fees and which article types are subject to charges, visit our website. If your manuscript is accepted for publication and any fees apply, you will be contacted separately about payment during the production process; please follow the instructions in that e-mail. Arrangements for payment must be made before your article is published.

ASM Membership: Corresponding authors may join or renew ASM membership to obtain discounts on publication fees. Need to upgrade your membership level? Please contact Customer Service at Service@asmusa.org.

The ASM Journals program strives for constant improvement in our submission and publication process. Please tell us how we can improve your experience by taking this quick Author Survey.

Thank you for submitting your paper to Spectrum.

Sincerely,
Gabriele Arcari
Editor
Microbiology Spectrum

Reviewer #1 (Comments for the Author):

Dear editor:
This study aimed to assess the burden of DTR-GNB colonization in ICU patients, explore its association with clinical outcomes and examine genomic similarities. The biggest drawback is that it is a single-center study. Some places should be addressed for this study.

1.As for the abstract, no related description of the Whole-genome sequencing result.
2.The p-value should be in italics.
3.As for Gram-negative bacteria: Escherichia coli, Klebsiella pneumoniae, Acinetobacter baumannii, and Pseudomonas aeruginosa, are all drugs used for DTR definition the same?
4.Line 216, colonization was defined as the presence of positive growth from the rectal swab culture. Are there any references to support this definition?
5.Data management and statistical analysis section, the p-value is not defined as being statistically significant.
6.When a bacterial name appears a second time, it is recommended to use the abbreviation, such as E. coli, K. pneumoniae, A. baumannii, and P. aeruginosa. The entire text should be checked.
7.No description of the ST strain or drug resistance genes was found.

Reviewer #2 (Comments for the Author):

Comments for the Author

This is an important and well-executed study that makes a meaningful contribution to the field. It provides strong genomic evidence supporting the link between gut colonization and subsequent infection in a high-burden ICU setting where data are often limited. The use of whole-genome sequencing, including ANI and SNP analysis, adds a high level of rigor and moves the work beyond standard phenotypic reporting.

The mortality findings are particularly striking. A 90.9% mortality rate among patients with DTR infections is a powerful result and deserves clear emphasis, especially in summaries or any broader dissemination of the work.

The way colonization is handled is also a strength. By distinguishing between patients who were colonized at enrollment and those who acquired colonization during their stay, the study clearly points to the ICU environment as a key site of transmission.

Suggestions for Improvement

There are a few areas where the manuscript could be strengthened further. Expanding the multivariable analysis would be helpful, particularly by adjusting for baseline illness severity using established scoring systems such as APACHE II or SOFA. This would make it clearer whether the observed differences in outcomes are directly attributable to DTR-GNB rather than underlying patient condition.

It would also be worth acknowledging the absence of environmental sampling as a limitation. Including a brief discussion on this point could help frame whether hospital acquisition is more likely driven by cross-transmission between patients or by environmental reservoirs.

The criteria used to select the 14 isolate pairs for sequencing should be clarified. A short explanation of whether these represent all available pairs or a selected subset would improve transparency.

A brief discussion on decolonization strategies could add value as well, even if only to outline the practical challenges of implementing such approaches in a high-pressure, resource-limited ICU setting.

Finally, consider adding a phylogenetic tree for the paired isolates. This would complement the ANI and SNP data and provide a more intuitive visual representation of the genomic relationships.

Remarks for the Editors

This study addresses a blind spot in global AMR surveillance. Data from tertiary care centers in Bangladesh is scarce, and the documented 90.9% mortality rate for DTR-GNB infections is a critical, high-impact finding that warrants urgent publication.

The integration of Whole-Genome Sequencing (WGS) to confirm clonality between rectal colonization and subsequent clinical infection moves the paper from descriptive to mechanistic, providing definitive evidence of the gut as a reservoir for sepsis in the ICU.

The acquisition rate (53% of patients negative at admission became colonized during their stay) is a damning indicator of local infection control breaches, making this a significant "real-world" study on the consequences of poor IPC (Infection Prevention and Control) in low-resource settings.

A minor concern for the editors is the use of selective chromogenic media. By focusing only on ESBL and carbapenemase producers, the authors cannot comment on the "fitness cost" or the competition between resistant and susceptible strains. However, this does not invalidate the primary findings regarding DTR pathogens.

The authors have clearly outlined their data sharing limitations via icddr,b protocols. While the clinical data is restricted, the editors should ensure the 28 genomic sequences are assigned accession numbers before final acceptance to maintain open-science standards.

The paper is highly suitable for journals focusing on clinical microbiology, infectious diseases, or hospital epidemiology, as it bridges the gap between laboratory genomics and bedside outcomes.

Comments for the Author

This is an important and well-executed study that makes a meaningful contribution to the field. It provides strong genomic evidence supporting the link between gut colonization and subsequent infection in a high-burden ICU setting where data are often limited. The use of whole-genome sequencing, including ANI and SNP analysis, adds a high level of rigor and moves the work beyond standard phenotypic reporting.

The mortality findings are particularly striking. A 90.9% mortality rate among patients with DTR infections is a powerful result and deserves clear emphasis, especially in summaries or any broader dissemination of the work.

The way colonization is handled is also a strength. By distinguishing between patients who were colonized at enrollment and those who acquired colonization during their stay, the study clearly points to the ICU environment as a key site of transmission.

Suggestions for Improvement

There are a few areas where the manuscript could be strengthened further. Expanding the multivariable analysis would be helpful, particularly by adjusting for baseline illness severity using established scoring systems such as APACHE II or SOFA. This would make it clearer

whether the observed differences in outcomes are directly attributable to DTR-GNB rather than underlying patient condition.

It would also be worth acknowledging the absence of environmental sampling as a limitation. Including a brief discussion on this point could help frame whether hospital acquisition is more likely driven by cross-transmission between patients or by environmental reservoirs.

The criteria used to select the 14 isolate pairs for sequencing should be clarified. A short explanation of whether these represent all available pairs or a selected subset would improve transparency.

A brief discussion on decolonization strategies could add value as well, even if only to outline the practical challenges of implementing such approaches in a high-pressure, resource-limited ICU setting.

Finally, consider adding a phylogenetic tree for the paired isolates. This would complement the ANI and SNP data and provide a more intuitive visual representation of the genomic relationships.

Response to Reviewers'

Reviewer 1:

This study aimed to assess the burden of DTR-GNB colonization in ICU patients, explore its association with clinical outcomes and examine genomic similarities. The biggest drawback is that it is a single-center study. Some places should be addressed for this study.

Dear Reviewer,

Thank you for your valuable time for reviewing our manuscript and your valuable comments. We've addressed these as follows. Also, we've now added our limitation of single-center study (Line no. 503-504 of track change version). We hope that this will be now suitable for your kind approval.

Comment 1. As for the abstract, no related description of the Whole-genome sequencing result.

Response: We thank the reviewer for this comment. We have now revised the abstract to explicitly describe the whole-genome sequencing analysis and its key findings as whole-genome sequencing (WGS) was performed on paired colonization and infection isolates and genomic relatedness was assessed using FastANI, core-genome SNP analysis and phylogenetic reconstruction. WGS revealed that 13 of 14 paired colonization-infection isolates were conspecific, with three pairs being highly clonal; whereas the remaining pairs showed greater genomic divergence, consistent with the SNP and phylogenetic analyses (Line no. 54-56 and 66-68 of track change version).

Comment 2. The p-value should be in italics.

Response: Thank you for finding this out. We've now italicized the p-value in both abstract and result sections (Line no. 64 and 340 of track change version).

Comment 3. As for Gram-negative bacteria: Escherichia coli, Klebsiella pneumoniae, Acinetobacter baumannii, and Pseudomonas aeruginosa, are all drugs used for DTR definition the same?

Response: Yes, antibiotic susceptibility of all Gram-negative bacteria was tested using the same Vitek 2 AST-N405 cards. So, all drugs used for DTR definitions are the same for all four Gram-negative bacteria in our study. We've now clarified these under Laboratory procedure and Operational definitions sections (Line no.: 172-173 and 227 of track change version).

Comment 4. Line 216, colonization was defined as the presence of positive growth from the rectal swab culture. Are there any references to support this definition?

Response: Thank you for let us clarifying the operational definition of colonization for our study. Several studies have used colonization from several samples such as rectal swab, stool, vaginal swab, skin, etc. In our study, we've used it for rectal swabs. I've now added two references of two recent studies that used rectal swabs to detect colonization. (Reference no. 17 and 18 in Line no. 228 of track change version).

Comment 5. Data management and statistical analysis section, the p-value is not defined as being statistically significant.

Response: Thank you for finding this missing information. We've now mentioned that considering 95% confidence interval, we've considered a $p\text{-value} < 0.05$ as statistically significant under the Data management and statistical analysis section (Line no. 244-245 of track change version).

Comment 6. When a bacterial name appears a second time, it is recommended to use the abbreviation, such as E. coli, K. pneumoniae, A. baumannii, and P. aeruginosa. The entire text should be checked.

Response: Thank you for your comment on formatting the scientific names. We've now updated these names throughout the manuscript.

Comment 7. No description of the ST strain or drug resistance genes was found.

Response: Thank you for this helpful comment. We agree that inclusion of sequence type and resistance gene information would strengthen the genomic characterization. We have revised the manuscript to describe the sequence types (STs) of the sequenced isolates under results section as "WGS further showed that the paired isolates belonged to diverse sequence types (STs). Among K. pneumoniae, several isolates belonged to ST11, whereas the A. baumannii isolates comprised multiple STs and novel profiles, highlighting genomic heterogeneity across paired colonization and infection isolates" (Line no. 361-364 of track change version).

All sequence types have also been added to the last column of Supplementary Table S1 (separate supplementary file).

With regard to resistance genes, this analysis was not part of the original study workflow. We have now acknowledged this limitation in the manuscript and plan to include resistance gene analysis in a forthcoming genomic study.

Reviewer 2:

This is an important and well-executed study that makes a meaningful contribution to the field. It provides strong genomic evidence supporting the link between gut colonization and subsequent infection in a high-burden ICU setting where data are often limited. The use of whole-genome sequencing, including ANI and SNP analysis, adds a high level of rigor and moves the work beyond standard phenotypic reporting.

The mortality findings are particularly striking. A 90.9% mortality rate among patients with DTR infections is a powerful result and deserves clear emphasis, especially in summaries or any broader dissemination of the work.

The way colonization is handled is also a strength. By distinguishing between patients who were colonized at enrollment and those who acquired colonization during their stay, the study clearly points to the ICU environment as a key site of transmission.

Dear Reviewer,

Thank you for reviewing our paper and your inspiring words and insightful suggestions that has improved the quality of this paper. We have tried to address your suggestions as below and hope that now it will be suitable for publication.

Suggestions for Improvement

Comment 1. There are a few areas where the manuscript could be strengthened further. Expanding the multivariable analysis would be helpful, particularly by adjusting for baseline illness severity using established scoring systems such as APACHE II or SOFA. This would make it clearer whether the observed differences in outcomes are directly attributable to DTR-GNB rather than underlying patient condition.

Response: Thank you for your kind suggestion. However, it is one of our limitations that we didn't collect the vitals and laboratory reports of the enrolled patients. So, we couldn't analyze the outcomes based on the illness severity scores. We've now added the following sentences as a limitation of our study.

"We did not collect detailed vital signs or laboratory data from the enrolled patients; therefore, we were unable to apply the illness severity scores like APACHE II or SOFA, or to perform multivariable analyses stratified by disease severity to assess mortality differences." (Line no. 504-507 of track change version).

Comment 2. It would also be worth acknowledging the absence of environmental sampling as a limitation. Including a brief discussion on this point could help frame whether hospital acquisition is more likely driven by cross-transmission between patients or by environmental reservoirs.

Response: We agree on this suggestion that our study could be improved more by testing samples from environment to know about the transmission dynamics. However, due to the budget constraint, we couldn't perform environmental sampling. Now we've added this under limitations as suggested (Line no. 507-510 of track change version).

Comment 3. The criteria used to select the 14 isolate pairs for sequencing should be clarified. A short explanation of whether these represent all available pairs or a selected subset would improve transparency.

Response: Thank you for letting us to give a brief explanation of tested samples in whole-genome sequencing. Yes, our 14 isolate pairs represent all available paired isolates. We've now explained this shortly under the Whole-Genome Sequencing (WGS) section of Methods as following:

"Among the 30 culture-confirmed infections, 13 patients had 14 paired isolates of bacterial species recovered from both clinical (blood, urine and tracheal aspirate) and rectal swab samples of the same patients. All of these available paired isolates underwent for whole-genome sequencing to detect the genomic similarities between colonized and infected strains found from the same patients." (Line no. 175-178 of track change version).

Comment 4. A brief discussion on decolonization strategies could add value as well, even if only to outline the practical challenges of implementing such approaches in a high-pressure, resource-limited ICU setting.

Response: Thank you for your kind suggestion. We've now updated the following sentences on decolonization strategies:

"Also, currently there is no conclusive and reliable evidence of decolonization practices, especially against Gram-negative bacteria in lower- and middle-income countries having limited resources. Further research should also be planned for evaluating the effectiveness of decolonization strategies such as selective digestive decontamination (SDD) or fecal microbiota transplantation (FMT) among such high-risk population with high death rate to reduce the morbidity and mortality related to multidrug-resistant pathogens." (Line no. 526-531 of track change version).

Comment 5. Finally, consider adding a phylogenetic tree for the paired isolates. This would complement the ANI and SNP data and provide a more intuitive visual representation of the genomic relationships.

Response: Thank you for your valuable suggestion to improve our paper. We've now added a phylogenetic tree for better visualization and easier understanding of the genomic relationship (Supplementary Figure no. 6 under separate file as supplementary file).

We've also included the findings of phylogenetic tree under results section as "Phylogenetic analysis was broadly concordant with the FastANI and SNP comparisons. Four species were represented among the paired isolate sets (*E. coli*, *A. baumannii*, *K. pneumoniae*, and *P. aeruginosa*), with one additional *Klebsiella quasipneumoniae* colonization isolate detected in one patient. Because only one paired isolate set was available for *E. coli* and *P. aeruginosa*, phylogenetic trees were generated only for *K. pneumoniae* and *A. baumannii*. In both species, infection and colonization isolates were interspersed across the trees rather than forming distinct status-specific clades, and pairs with higher ANI values and lower SNP distances clustered more closely than more divergent pairs, indicating heterogeneous genomic relatedness between paired colonization and infection isolates. The *K. pneumoniae* colonization isolate from one patient was excluded from the phylogeny because it was identified as *K. quasipneumoniae*." (Line no. 391-401 of track change version).

We've described the findings too under the discussion section as "The phylogenetic trees were broadly consistent with the FastANI and SNP analyses and provided a complementary visual framework for interpreting genomic relatedness among paired isolates. Importantly, the phylogenetic reconstruction did not show clear separation by clinical status, as infection and colonization isolates were interspersed across the trees rather than forming distinct clades. Taken together, the concordance between FastANI, SNP-based comparisons and phylogenetic analysis supports the interpretation that the genomic relationship between colonization and infection isolates is heterogeneous, with some pairs being closely related and others representing genetically distinct lineages." (Line no. 482-490 of track change version).

Re: Spectrum00121-26R1 (Difficult-to-treat Resistant Gram-negative Bacteria and Genomic Resemblances between Colonization and Infection among Patients in an Intensive Care Unit of a Tertiary Care Hospital in Bangladesh)

Dear Dr. Gazi Md. Salahuddin Mamun:

Your manuscript has been accepted, and I am forwarding it to the ASM production staff for publication. Your paper will first be checked to make sure all elements meet the technical requirements. ASM staff will contact you if anything needs to be revised before copyediting and production can begin. Otherwise, you will be notified when your proofs are ready to be viewed.

Data Availability: ASM policy requires that data be available to the public upon online posting of the article, so please verify all links to sequence records, if present, and make sure that each number retrieves the full record of the data. If a new accession number is not linked or a link is broken, provide production staff with the correct URL for the record. If the accession numbers for new data are not publicly accessible before the expected online posting of the article, publication may be delayed; please contact ASM production staff immediately with the expected release date.

Publication Fees: For information on publication fees and which article types have charges, please visit our website. We have partnered with Copyright Clearance Center (CCC) to collect author charges. If fees apply to your paper, you will receive a message from no-reply@copyright.com with further instructions. For questions related to paying charges through RightsLink, please contact CCC at ASM_Support@copyright.com or toll free at +1-877-622-5543. CCC makes every attempt to respond to all emails within 24 hours.

ASM Membership: Corresponding authors may join or renew ASM membership to obtain discounts on publication fees. Need to upgrade your membership level? Please contact Customer Service at Service@asmusa.org.

Embargo Policy: A press release may be issued as soon as the manuscript is posted on the Spectrum Latest Articles webpage. The corresponding author will receive an email with the subject line "ASM Journals Author Services Notification" when the article is available online.

The ASM Journals program strives for constant improvement in our submission and publication process. Please tell us how we can improve your experience by taking this quick Author Survey.

Thank you for submitting your paper to Spectrum.

Sincerely,
Gabriele Arcari
Editor
Microbiology Spectrum
