# Supplementary material for: Difficult-to-treat resistant Gram-negative bacteria and genomic resemblances between colonization and infection among patients in an intensive care unit of a tertiary care hospital in Bangladesh
Source: Microbiol Spectr. 2026 Jul 23;14(9):e00121-26. doi: 10.1128/spectrum.00121-26 (PMC13532269; doi:10.1128/spectrum.00121-26)
Supplement: Supplementary file 2 [file spectrum.00121-26-s0001.docx]

**Supplementary files:**

**Supplementary Figure S1. Distribution of enrolled patients colonized with Gram-negative bacteria** (n=373). The horizontal bars show the numbers of Gram-negative bacteria detected from rectal swabs. The vertical bars show the co-colonization with these GNBs.

**Supplementary Figure S2. Resistance pattern against tested antibiotics of colonized *E. coli* isolates (n=502)**

**Supplementary Figure S3. Resistance pattern against tested antibiotics of colonized *K. pneumoniae* isolates (n=280)**

**Supplementary Figure S4. Resistance pattern against tested antibiotics of colonized *A. baumannii* isolates (n=152)**

**Supplementary Figure S5. Resistance pattern against tested antibiotics of colonized *P. aeruginosa* isolates (n=26)**

**Supplementary Figure S6. Species-specific phylogenetic trees of paired colonization and infection isolates.** Maximum-likelihood phylogenies were reconstructed from species-level core-genome alignments for (A) *K. pneumoniae* and (B) *A. baumannii*. Tip labels indicate patient ID and clinical status. Infection isolates are shown in red and colonization isolates in blue, and the adjacent color strip indicates patient identity. Branch support values are shown on the internal nodes.

**Supplementary Table S1.** Genomic and clinical information of 13 patients with 14 paired colonization and infection isolates following whole-genome sequencing.

| **ID** | **Status (Colonization/ Infection)** | **Coverage (x)** | **Genome Length (bp)** | ***Species*** | **ST Type** |
| --- | --- | --- | --- | --- | --- |
| 1007 | Colonization | 59.98 | 5361985 | *Escherichia coli* | ST2083 |
|  | Infection | 59.08 | 5433199 | *E. coli* | ST448 |
| 1026 | Colonization | 52.61 | 5489348 | *Klebsiella quasipneumoniae* | ST3880 |
|  | Infection | 44.7 | 5712896 | *Klebsiella pneumoniae* | ST11 |
| 1059 | Infection | 54.07 | 5605584 | *K. pneumoniae* | ST709 |
|  | Colonization | 57.07 | 5532422 | *K. pneumoniae* | ST437 |
| 1081 | Colonization | 49.13 | 5698681 | *K. pneumoniae* | ST395 |
|  | Infection | 51.67 | 5840524 | *K. pneumoniae* | ST11 |
| 1270 | Infection | 75.85 | 4002177 | *Acinetobacter baumannii* | ST2441 |
|  | Colonization | 67.7 | 5237969 | *A. baumannii* | ST848,ST1956-PARALOG |
| 1278 | Infection | 84.31 | 3917086 | *A. baumannii* | Novel_profile |
|  | Colonization | 84.37 | 3914043 | *A. baumannii* | Novel_profile |
| 1279 | Colonization | 100.4 | 3918267 | *A. baumannii* | ST1418 |
|  | Infection | 97.57 | 3969412 | *A. baumannii* | ST2275 |
| 1293 | Colonization | 52.4 | 5928893 | *K. pneumoniae* | ST3130 |
|  | Infection | 44.19 | 5737870 | *K. pneumoniae* | ST147 |
| 1328 | Colonization | 51.76 | 5798013 | *K. pneumoniae* | ST11 |
|  | Infection | 46.85 | 5684655 | *K. pneumoniae* | ST11 |
| 1343 | Infection | 38.65 | 5683594 | *K. pneumoniae* | ST11 |
|  | Colonization | 58.32 | 5715822 | *K. pneumoniae* | Novel_profile |
| 1349_a | Colonization | 107.9 | 3741203 | *A. baumannii* | Novel_allele |
|  | Infection | 103.8 | 3947359 | *A. baumannii* | ST848,ST1956-PARALOG |
|  | Infection | 86.04 | 3946295 | *A. baumannii* | ST848,ST1956-PARALOG |
|  | Colonization | 97.05 | 3740256 | *A. baumannii* | Novel_allele |
|  | Infection | 102.7 | 3945505 | *A. baumannii* | ST848,ST1956-PARALOG |
| 1349_p | Colonization | 54.52 | 6782181 | *Pseudomonas aeruginosa* | ST357 |
|  | Infection | 58.12 | 6741750 | *P. aeruginosa* | ST357 |
| 1366 | Colonization | 48.48 | 5684435 | *K. pneumoniae* | ST11 |
|  | Infection | 54.55 | 5842391 | *K. pneumoniae* | ST11 |
| 1371 | Infection | 42.02 | 5684176 | *K. pneumoniae* | ST11 |
|  | Colonization | 56.39 | 5716011 | *K. pneumoniae* | Novel_profile |

**Supplementary Table S2.** Comparative genomic analysis of paired infection and colonization isolates based on whole-genome sequencing

| Sample | Species | Average Nucleotide Identity | Core SNP* |
| --- | --- | --- | --- |
| ‍1349_a | *Acinetobacter baumannii* | 99.9977 | 7 |
| 1278 | *A. baumannii* | 99.999 | 9 |
| 1349_p | *Pseudomonas aeruginosa* | 99.9991 | 29 |
| 1328 | *Klebsiella pneumoniae* | 99.947 | 219 |
| 1366 | *K. pneumoniae* | 99.945 | 251 |
| 1007 | *Escherichia coli* | 100 | 13,790 |
| 1081 | *K. pneumoniae* | 99.358 | 15,780 |
| 1343 | *K. pneumoniae* | 98.853 | 29,865 |
| 1371 | *K. pneumoniae* | 98.857 | 30,175 |
| 1059 | *K. pneumoniae* | 98.904 | 32,908 |
| 1293 | *K. pneumoniae* | 98.888 | 34,581 |
| 1279 | *A. baumannii* | 97.771 | 57,915 |
| 1270 | *A. baumannii* | 97.854 | 58,338 |
| 1026 | *K. pneumoniae* and *K.* *quasipneumoniae* | 94.021 | 229,309 |

* SNP: Single Nucleotide Polymorphism
